# Effects of dietary nucleotide supplementation on growth performance, immune function, and intestinal microbiota in weaned kittens

**DOI:** 10.3389/fvets.2026.1843322

**Published:** 2026-06-18

**Authors:** Can Zhang, Lupeng Qin, Zhanzhao Zhao, Xueqi Yuan, Jingtong Kang, Bin Li, Jianguo Zhang, Shijian Nie

**Affiliations:** China Pet Foods Pet Nutrition and Health Research Institute, China Pet Technology (Yantai) Co., Ltd., Yantai, China

**Keywords:** growth hormones, growth performance, immune function, intestinal microbiota, nucleotides, weaned kittens

## Abstract

**Introduction:**

This study aimed to investigate the effects of yeast-derived nucleotides on growth performance, serum biochemical indices, apparent digestibility, growth hormones, immune function, and intestinal microbiota in weaned kittens.

**Methods:**

Sixteen 4-month-old healthy weaned kittens were randomly assigned into two groups with 8 kittens each. The control group (CON) was fed a basal dry food diet, the experimental group (TREAT) was fed the basal dry food diet supplemented with 0.03% yeast-derived nucleotides.

**Results:**

After 42 days of feeding, the levels of total protein (TP), albumin (ALB), globulin (GLB), creatinine (Crea) and aspartate aminotransferase (AST) were significantly increased (*p* < 0.05) in TREAT group. The apparent digestibility of dry matter (DM), organic matter (OM), and crude protein (CP) in the TREAT group was significantly higher than that in the CON group (*p* < 0.05). The serum concentrations of growth hormone (GH), insulin-like growth factor-1 (IGF-1), and immunoglobulin G (IgG) in the TREAT group were significantly increased (*p* < 0.01) and were significantly higher than CON group (*p* < 0.01). The concentrations of immunoglobulin A (IgA) and immunoglobulin M (IgM) were significantly higher than CON group (*p* < 0.01). At the genus level, compared with the CON group, the relative abundances of Sellimonas and *Acutalibacter* in the TREAT group were significantly increased (*p* < 0.05), while those of *Lachnospiraceae_NK4A136_group* and *Sarcina* were significantly decreased (*p* < 0.05).

**Discussion:**

In conclusion, dietary supplementation with 0.03% yeast-derived nucleotides improved the nutrient utilization efficiency and presents a potential to maintain relatively higher humoral immunity and improve health condition of weaned kittens.

## 1 Introduction

Nucleotides (NT) are important small-molecule bioactive compounds in animal organisms. They not only serve as the fundamental structural moieties of deoxyribonucleic acid (DNA) and ribonucleic acid (RNA), but also act as key components of numerous coenzymes, exerting a crucial regulatory role in a variety of metabolic pathways. Nucleotides are involved in almost all biological processes including cellular energy metabolism, signal transduction, and gene expression and regulation. Under normal physiological conditions, animals can synthesize nucleotides through endogenous metabolic pathways, thus nucleotides are classified as non-essential nutrients for canines and felines (1). However, in specific physiological states with vigorous metabolic demands, endogenous nucleotide synthesis is insufficient to meet the body physiological requirements. For instance, during the growth and development stage, pregnancy and lactation periods, or under stress conditions and intestinal mucosal damage, exogenous nucleotide supplementation is required to satisfy the body metabolic requirements (2, 3). The nucleotide content varies significantly among different food sources: visceral organs, yeast, and seafood are rich in nucleic acids, with a nucleic acid content of 100–1,000 mg per 100 g of fresh sample; meat and mushrooms have relatively low nucleic acid contents, with 90–100 mg per 100 g of fresh sample (4).

Previous studies have demonstrated that dietary nucleotide supplementation is conducive to enhancing humoral and cellular immune functions in weaned single-stomached and exerts a positive effect on their overall health status (5, 6). Romano et al. (7) reported that Beagle puppies supplemented with dietary nucleotides exhibited a more robust humoral immune response compared with the control group, with significantly increased serum immunoglobulin levels (IgG, IgA, and IgM). In addition, supplementing infant formula with nucleotides at a proportion similar to that in breast milk results in consistent weight and length growth between formula-fed and breastfed infants, and can improve infant immune function and plasma lipid parameters, leading to the widespread application of nucleotides in infant formula production (8).

Weaned young animals are in a stage of rapid growth and development (9), yet their immune system is not fully mature and immune function is relatively low (10). Therefore, nucleotide supplementation can better promote the growth and development of weaned young animals and enhance their immune competence (5). This study aimed to investigate the effects of yeast-derived nucleotides on growth performance, apparent nutrient digestibility, growth hormone levels, immune function, and intestinal microbiota in weaned kittens, with the purpose of providing basic experimental data and theoretical support for the formulation design of specialized feed for weaned kittens.

## 2 Materials and methods

### 2.1 Animals

The experimental protocol was approved by the Pet Welfare and Ethics Committee of China Pet Technology (Yantai) Co., Ltd. (protocol number: CPPWEF-2025-011).

Sixteen 4-month-old weaned kittens, six male and ten female of five different breeds (two Ragdoll, two Devon, two Maine, four American short hair and six British short hair kittens) weighing 1.7–3.4 kg, which were clinically diagnosed as healthy by a licensed veterinarian, were used in this study as experimental animals. Kittens were housed in two catteries (ten square meters of each) in a natural light and temperature environment, and were fed individually in cages (1.6 m × 0.7 m × 0.6 m) of each cattery. All kittens allowed to come out of their cages move freely from 11:00 a.m. to 14:00 p.m.

### 2.2 Experimental design and diets

The kittens were randomized to control group (CON, *n* = 8) and the test group (TREAT, *n* = 8), balanced for sex, genetics (through equal distribution of kittens from the same breed across groups). The CON kittens received an extruded dry food diet; the TREAT kittens received the same diet supplemented with 3 g yeast-derived nucleotides per kg of diet. Nucleotide mixture (Angel Yeast Ltd., Yichang, China) analyzed composition (g/kg dry matter): crude protein: 548; total nucleic acids: 126; amino acid nitrogen: 29; ash: 78. Water content: 33 g/kg as-is.

All experimental kittens had free access to clean drinking water and diets ad libitum throughout the trial period. The pre-feeding adaptation period lasted for 7 days, and the formal feeding trial was conducted for 42 days. All kittens were immunized with the feline rhinotracheitis-calici-panleukopenia vaccine (killed virus, Zoetis, USA) before the experiment (−1d) and received subsequent two vaccinations every 28 days after the first vaccination. The basal diet was formulated in accordance with the nutritional requirements for kittens specified by the Association of American Feed Control Officials (11). The composition and nutritional levels of the basal diet are presented in Table 1. The contents of crude protein, ether extract, ash, and moisture were determined in accordance with the recommended methods of the Association of Official Analytical Chemists (AOAC). The metabolizable energy (ME) of the diet was calculated with reference to the Nutrient Requirements of Dogs and Cats published by National Research Council (1).

**Table 1:** Composition and nutrient levels of study diets (dry matter basis).

Ingredients, % DM		Nutrient composition, % DM	
Frozen chicken	5.00	ME^2^ kcal/kg	4,241
Frozen duck	5.00	Crude protein (CP), %	33.5
Chicken meal	15.00	Ether extract (EE), %	12.5
Chicken liver	5.00	Crude fiber (CF), %	4.0
Chicken heart	2.00	Ash, %	9.0
Salmon	1.00	Calcium (Ca), %	1.41
Beet pulp	5.00	Phosphorus (P), %	1.27
Corn	24.00		
Wheat	14.50		
Pea	5.00		
Potato starch	3.00		
Beef fat	1.00		
Fish oil	0.50		
Chicken fat	8.00		
Beer yeast powder	2.00		
Calcium carbonate	0.60		
Yucca powder	0.10		
Blueberry powder	0.40		
Cranberry powder	0.30		
Tomato powder	0.60		
Premix^1^	2.00		

1 Formulated to provide the following nutrients per kilogram of premix: Vitamin A 800000 IU, Vitamin E 5700 IU, Vitamin B1 620 mg, Vitamin D3 1800000 IU, Vitamin B2 850 mg, Vitamin K3 90 mg, Vitamin C 38000 mg, Niacin 4,200 mg, D-Pantothenic Acid 75 mg, Choline 35 g, Fe 8,000 mg, Cu 1,000 mg, Mn 1,000 mg, Zn 4,800 mg, I 55 mg, Se 18 mg, Co 53 mg.

2 Metabolizable energy was a calculated value.

### 2.3 Sampling and measurement

#### 2.3.1 Growth performance

The body weight of experimental kittens was measured in the fasting state on day 0, 14, 28, and 42 of the formal experiment. Body condition score (BCS) was evaluated with reference to the World Small Animal Veterinary Association (WSAVA) Cat Body Condition Score System (9-point system). The daily feed intake and residual feed amount were accurately recorded during the experiment to calculate the average dry matter intake (DMI).

#### 2.3.2 Fecal score

Fecal samples of experimental kittens were monitored and collected daily throughout the feeding trial. Fecal scoring was performed in a familiar environmental context for the kittens with reference to The WALTHAM™ Faeces Scoring System (Table 2).

**Table 2:** The WALTHAM™ faeces scoring system.

Score	Standard
1	“Bullet-like,” breaks apart with slight pressure
1.5	Hard and dry feces, cracks when pressed
2	Well-formed, leaves no residue when picked up
2.5	Well-formed, slightly moist surface, leaves slight residue when picked up
3	Moist and somewhat loose feces, leaves clear residue when picked up
3.5	Very moist but still retains a definite shape
4	Sticky liquid feces with some solids in the liquid
4.5	Slightly sticky liquid feces
5	Watery liquid feces

#### 2.3.3 Nutrient apparent digestibility

During the final 7 days of the formal feeding trial, each kitten was individually fed in a cage. Fecal samples were collected by using transparent resinous granules (4.35 mm × 3.35 mm × 2.85 mm of each) litter and thoroughly homogenized, portion was combined with an equal volume of 10% dilute sulfuric acid for nitrogen fixation. All fecal samples were stored at −20 °C. Feed samples were also collected and thoroughly homogenized, and subjected to the quartering method for representative sampling. After the feeding trial, both fecal and feed samples were dried in a forced-air drying oven at 65 °C for 48 h. Subsequently, the dried samples were ground using a Cyclotec mill (Tecator 1,093; Tecator AB, Höganäs, Sweden) equipped with a 40-mesh sieve for conventional nutrient component analysis. A portion of the air-dried feed samples was further dried at 105 °C for 3 h to determine the dry matter content. Diet and feces were analyzed for organic matter (OM), crude protein (CP), ether extract (EE), and ash in using the AOAC official methods (12). The apparent nutrient digestibility was calculated using acid-insoluble ash (AIA) as an internal marker, following the method established by Van Keulen and Young (13). The calculation formula is as follows:

Nutrient digestibility (%) = [1 - (AIA concentration in feed / AIA concentration in feces) × (Nutrient concentration in feces / Nutrient concentration in feed)] × 100%.

#### 2.3.4 Blood indices

On the 0 and 42nd day of the formal experimental period, blood samples were collected from the jugular vein using a vacuum tube pre- morning feeding. After natural clotting, the blood samples were centrifuged at 3500 × g for 10 min at 4 °C to separate serum; the obtained serum samples were immediately stored in liquid nitrogen for subsequent analysis of aspartate aminotransferase (AST), alanine aminotransferase (ALT), and alkaline phosphatase (ALP), total protein (TP), albumin (ALB), Urea, creatinine (Crea), creatine kinase (CK), glucose (GLU), total cholesterol (TCHO), and triglycerides (TG). Serum biochemical indices were measured using the Catalyst One automatic biochemical analyzer (IDEXX, Westbrook, ME, United States). The globulin concentration (GLB) was calculated by subtracting the albumin concentration from the total protein concentration. Growth hormone (GH, Catalog Number: ml933652A), insulin-like growth factor-1 (IGF-1, Catalog Number: ml933025A), immunoglobulin (IgA, Catalog Number: ml952226A; IgG, Catalog Number: ml952225A; IgM, Catalog Number: ml277458A) were analyzed by using feline-specific ELISA assay kits (mlbio, Shanghai, China). Firstly, add 100 μL of standard solutions in different concentrations and test samples to each well individually, cover with sealing tape and react at 37 °C in the dark for 1.5 h. After incubation, 300 μL washing buffer was added to each well, shake well and pat it dry, repeat washing process three times. Then, add 100 μL of biotinylated antibody solution to each well, gently mix well, and cover with sealing tape react at 37 °C in the dark for 1 h. After the incubation, repeat the washing process four times, and add 100 μL of 1 × SA-HRP solution to each well and cover with sealing tape. React at 37 °C in the dark for 30 min, wash four times, and pat dry. Secondly, add 50 μL of chromogenic solution A to each well, followed by 50 μL of chromogenic solution B, mix well, cover with sealing tape, and react at 37 °C in the dark for 15 min. When reaction is completed, add 50 μL of the stop solution to each well and mix well. The absorbance values of each well were measured at 450 nm within 5 min by a Biotek ELX808 microplate absorbance reader (USA).

#### 2.3.5 Fecal microbiota

Total microbial genomic DNA was extracted from fecal samples using the E. Z. N. A.^®^ Soil DNA Kit (Omega Bio-tek, Norcross, GA, USA) in strict accordance with the manufacturer operating instructions. The integrity and concentration of the extracted genomic DNA were evaluated by 1.0% agarose gel electrophoresis and a NanoDrop^®^ ND-2000 ultraviolet–visible spectrophotometer (Thermo Scientific, USA), respectively; the qualified DNA extracts were stored at −80 °C until subsequent molecular biological analysis. For amplicon high-throughput sequencing, different specific primer sets were used for the amplification of bacteria. The V3–V4 hypervariable region of the bacterial 16S rRNA gene was amplified with the specific primers forward (5′-ACTCCTACGGGAGGCAGCA-3′) and reverse (5′-GGACTACHVGGGTWTCTAAT-3′). Polymerase chain reaction (PCR) amplification and amplicons were purified by magnetic bead purification, and the concentration of purified amplicons was quantified with an ABI Veriti 96-well 9,902 fluorometer (Applied Biosystems, Foster City, CA, USA). Microbial high-throughput sequencing was performed by Beijing Auwigene Gene Technology Co., Ltd. on an Illumina novaseq6000 platform with paired-end sequencing mode.

Raw sequencing reads were subjected to quality control and filtering, and valid reads were merged using PEAR software (v0.9.6). Sequences with a quality score below 20, ambiguous bases, or primer mismatches were excluded from subsequent analysis. During the sequence merging process, the minimum overlap length was set to 10 bp, and the maximum mismatch rate was set to 0.1. The merged sequences were further filtered using Vsearch software (v2.7.1) to remove short sequences with a length of less than 230 bp. Chimeric sequences were identified and eliminated using the UCHIME algorithm against the Gold Database. High-quality clean sequences were clustered into operational taxonomic units (OTUs) using the UPARSE algorithm implemented in Vsearch software (v2.7.1) at a 97% sequence similarity threshold (14). Representative sequences of each OTU were taxonomically annotated using the RDP Classifier algorithm against the Silva 128 database with a 70% confidence threshold (15). Alpha diversity indices including the Shannon index, Simpson index, and Chao1 index were calculated using QIIME2 software. Based on the taxonomic annotation results and relative abundance data, bar plots of microbial community composition were generated using R software (v3.6.0). Beta diversity distance matrices were calculated in QIIME software (v1.8.0), and principal coordinate analysis (PCoA) was performed based on weighted UniFrac distances using R software (v3.6.0).

### 2.4 Statistical analysis

The indices of growth performance, apparent nutrient digestibility, and serum biochemical parameters of kittens were statistically analyzed using SPSS 22.0 software. Paired t-tests were used for comparisons at different time points within groups, and Student’s *t*-tests were used for comparisons at same time points between groups. Differences in alpha diversity indices and the relative abundances of microbial flora between the two groups were analyzed using nonparametric Kruskal-Wallis tests. Spearman’s rank correlation coefficients between the relative abundances of all pairwise microbial genera were calculated using the Hmisc package in R software (v3.6.0). All statistical tests were performed at a significance level of *p* < 0.05.

## 3 Results

### 3.1 Growth performance and fecal score

No significant difference was observed in the DMI and ADG of two groups (*p* > 0.05). The final body condition score and fecal score of the two groups were at a normal physiological level. The fecal score was significantly lower in TREAT group compared to CON group (*p* < 0.05) (Table 3).

**Table 3:** Effects of dietary nucleotide supplementation on the growth performance and fecal score of kittens.

Item	Groups		SEM	*p* value
	CON	TREAT		
Dry matter intake (g)	57.07 ± 1.24	59.34 ± 1.39	1.86	0.226
Initial body weight (kg)	2.23 ± 0.09	2.31 ± 0.13	0.16	0.544
Final body weight (kg)	2.74 ± 0.15	2.91 ± 0.16	0.22	0.622
Final body condition score	4.88	5.13	0.18	0.179
Fecal score	2.74^*^	2.67	0.03	0.010

CON (Control diet), TREAT (Containing 0.03% yeast-derived nucleotides).

### 3.2 Nutrient apparent digestibility

The apparent digestibility of DM, OM, and CP in the TREAT group was significantly higher compared to the CON group (*p* < 0.01). The apparent digestibility of EE in the TREAT group was significantly higher compared to the CON group (*p* < 0.05) (Table 4).

**Table 4:** Effects of dietary nucleotide supplementation on the nutrient apparent digestibility of kittens.

Item	Groups		SEM	*P* value
	CON	TREAT		
Dry Matter (DM, %)	78.75	82.63^***^	0.81	<0.001
Organic Matter (OM, %)	83.52	86.58^**^	0.74	0.001
Crude Protein (CP, %)	84.53	87.60^**^	0.85	0.003
Ether Extract (EE, %)	91.29	93.80^**^	0.90	0.014

CON (Control diet), TREAT (Containing 0.03% yeast-derived nucleotides).

### 3.3 Serum biochemical indices

In comparison to the 0d, the levels of TP, ALB, GLB, Crea and AST were significantly increased (*p* < 0.05) at the end of the experiment in TREAT group. No significant differences were observed between two groups for other indices, including AST, ALT, ALP, TP, ALB, GLB, Urea, Crea, CK, GLU, TCHO, and TG (*p* > 0.05) (Table 5).

**Table 5:** Effects of dietary nucleotide supplementation on serum biochemical indices of kittens.

Items	Groups								Time			
	0d		SEM	*P* value	42d		SEM	*p*value	con		treat	
	CON	TREAT			CON	TREAT			SEM	*P*value	SEM	*P*value
TP, g/L	66.83	67.81	3.18	0.764	71.23	76.40^**^	3.03	0.113	2.08	0.079	1.84	0.003
ALB, g/L	29.44	29.59	0.86	0.871	30.94	32.04^**^	1.18	0.369	1.08	0.213	0.56	0.005
GLB, g/L	37.39	38.23	3.07	0.790	40.29	44.36^*^	3.27	0.237	1.96	0.190	1.85	0.016
A/G	0.81	0.79	0.07	0.684	0.79	0.76	0.06	0.636	0.05	0.604	0.03	0.356
UREA, mmol/L	8.82	10.18	0.65	0.058	9.61	10.68	0.52	0.071	0.33	0.056	0.82	0.569
Crea, umol/L	77.04	79.04	9.17	0.831	78.11	92.80^*^	6.78	0.051	6.13	0.867	4.59	0.024
CK, U/L	161.69	123.94	25.88	0.170	171.10	177.84	31.48	0.834	34.16	0.792	24.45	0.070
TCHO, mmol/L	3.58	4.04	0.30	0.150	3.17	4.05	0.46	0.080	0.37	0.313	0.42	0.982
TG, mmol/L	0.50	0.60	0.19	0.610	0.72	1.07	0.25	0.183	0.13	0.147	0.30	0.167
GLU, mmol/L	4.63	4.71	0.23	0.720	4.67	4.79	0.21	0.570	0.22	0.869	0.16	0.654
ALP, U/L	88.50	66.37	20.49	0.301	96.20	72.39	14.73	0.132	11.79	0.538	5.48	0.315
ALT, U/L	55.53	45.20	9.77	0.311	59.77	56.50	14.47	0.825	5.33	0.457	9.72	0.289
AST, U/L	24.39	20.03	2.23	0.089	27.43	26.64^*^	3.89	0.871	1.07	0.030	2.43	0.035

CON (Control diet), TREAT (Containing 0.03% yeast-derived nucleotides).

### 3.4 Growth hormones

**Figure 1:**
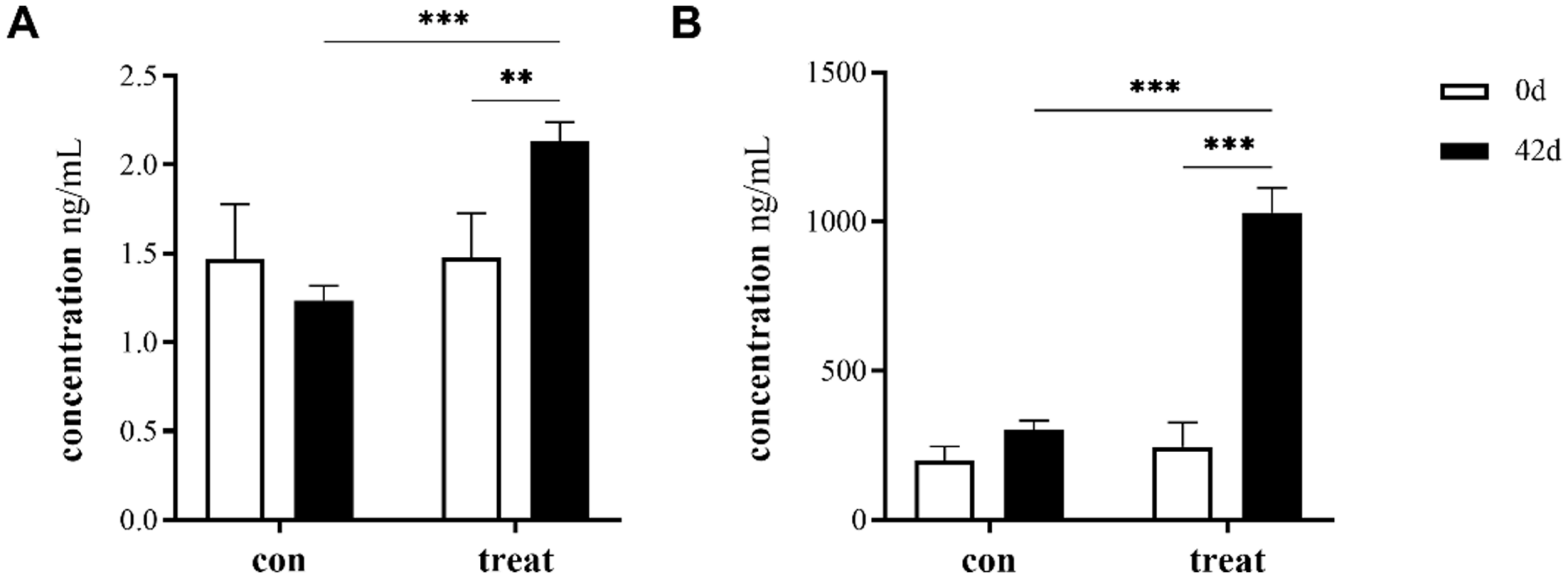
Effects of dietary nucleotide supplementation on serum growth factors in kittens. **(A)** Growth Hormone. **(B)** Insulin-like growth factor 1.

No significant differences were observed in serum GH and IGF-1 concentrations between two groups (*p* > 0.05) at 0d. In comparison to the 0d, the serum concentrations of GH and IGF-1 were significantly increased (*p* < 0.01) in TREAT group at 42d. After 42 days of dietary treatment, serum concentrations of GH and IGF-1 in the TREAT group were significantly higher compared to the CON group (*p* < 0.001) (Figure 1).

### 3.5 Immune indices

**Figure 2:**
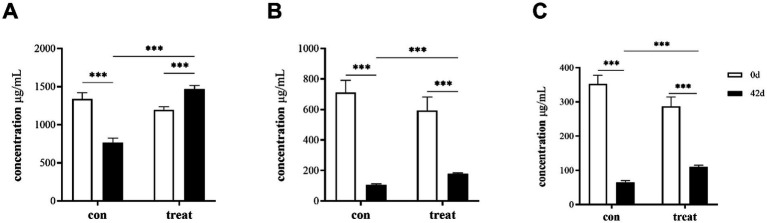
Effects of dietary nucleotide supplementation on serum immune indices in kittens **(A)** Immunoglobulin G, **(B)** Immunoglobulin A, **(C)** Immunoglobulin M.

There was no significant statistical difference in serum IgG, IgA and IgM concentration between the TREAT group and the CON group (*p* > 0.05) at 0d. After 42 days of dietary treatment, the serum IgG concentration in the TREAT group were significantly increased compared to the initial level (*p* < 0.01), and significantly higher compared to the CON group (*p* < 0.01). After 42 days of dietary treatment, the serum IgA and IgM concentrations in the TREAT group were significantly higher compared to the CON group (*p* < 0.01), and they were significantly decreased compared to the initial levels (*p* < 0.01) (Figure 2).

### 3.6 Fecal microbial diversity

**Figure 3:**
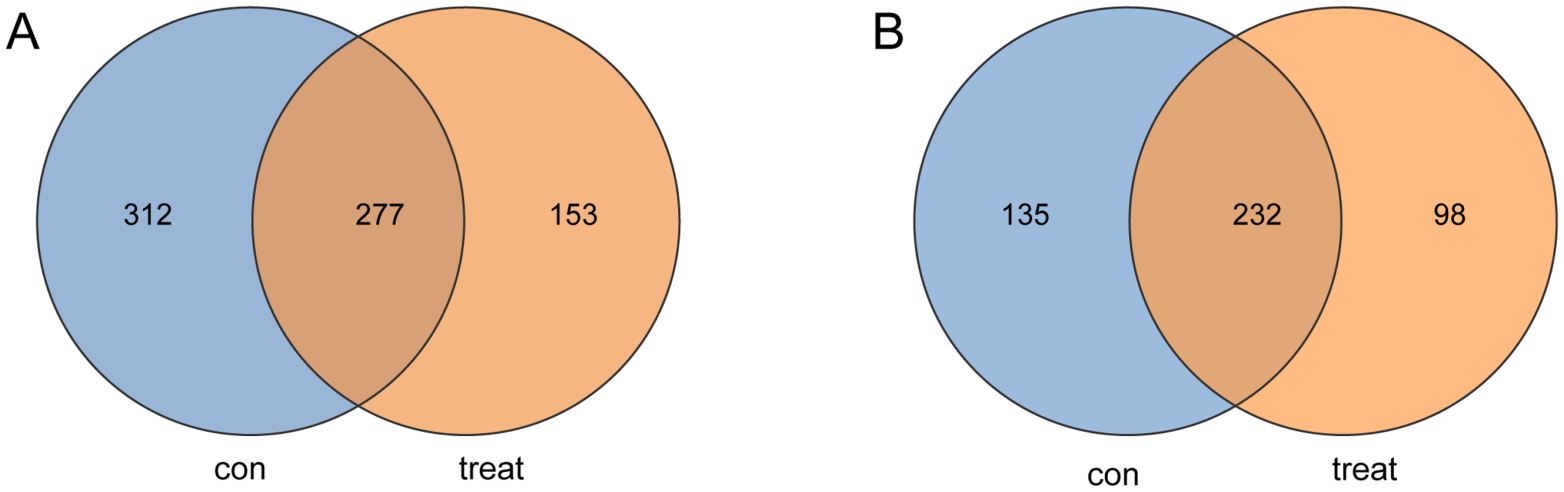
Effects of dietary nucleotide supplementation on the composition of core gut microbiota in kittens: **(A)** Before dietary treatment, **(B)** After dietary treatment.

There were 312 specific OTUs in the CON group, and 153 specific OTUs in the TREAT group before dietary treatment. In addition, 277 core OTUs were shared in both groups (Figure 3A). After 42 days of dietary treatment, the number of specific OTUs decreased to 135 and 98 in CON and TREAT group, respectively. Besides, the shared core OTUs in both groups were decreased to 232 (Figure 3B).

**Figure 4:**
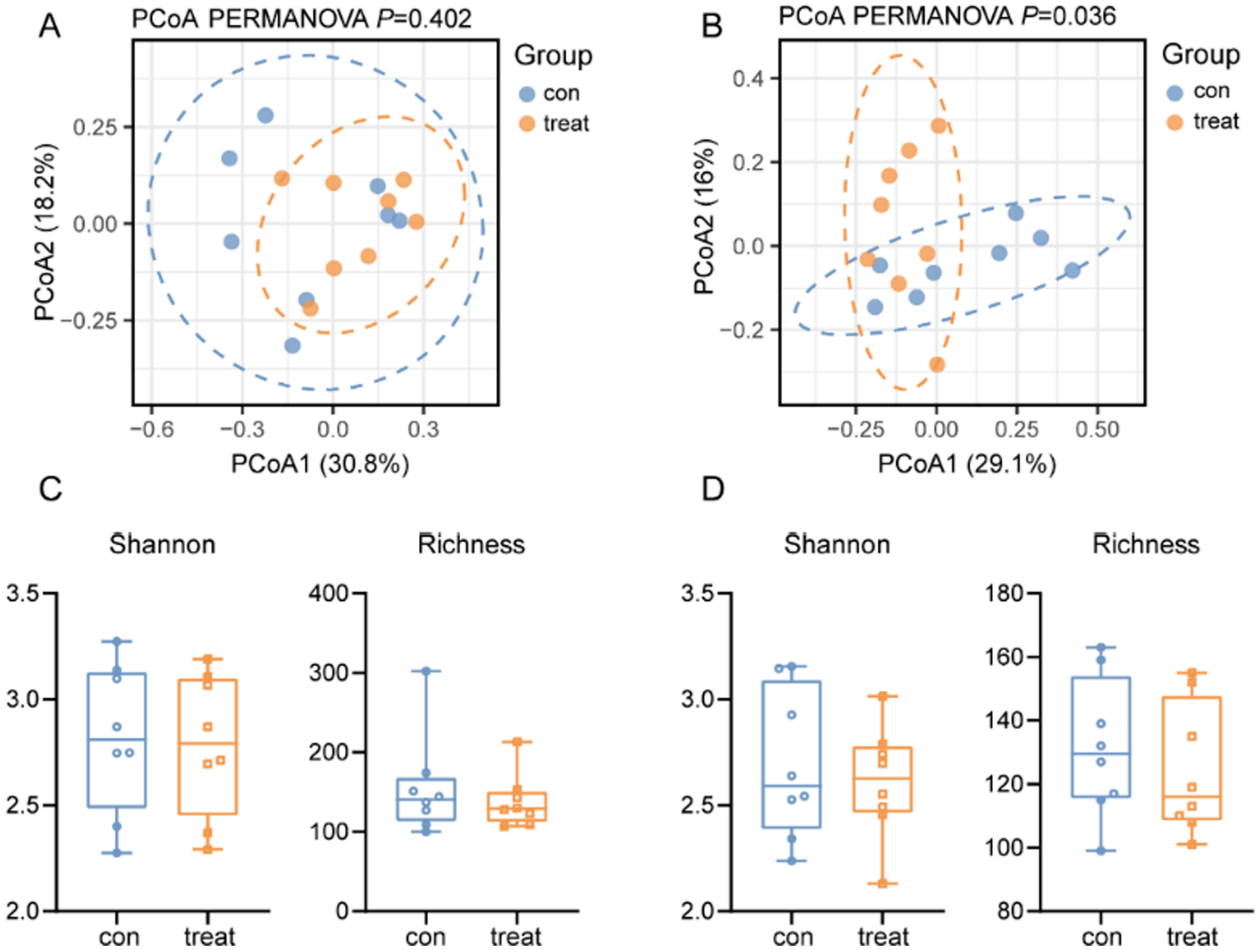
Effects of dietary nucleotide supplementation on intestinal bacterial diversity in kittens. **(A,C)** Before dietary treatment, **(B,D)** After dietary treatment.

As presented in Figures 4A,C, principal coordinate analysis (PCoA) based on weighted UniFrac distances showed that there was no significant difference in the overall structure of the fecal microbial community between two groups before dietary treatment (*p* > 0.05). The results of alpha diversity analysis indicated that the Shannon index and species richness values of the two groups were similar. As presented in Figures 4B,D, after 42 days of dietary treatment, there was a significant difference in the overall structure of the fecal microbial community between the two groups (*p* < 0.05); the Shannon index and species richness of the TREAT group were lower than those of the CON group.

### 3.7 Fecal microbial differences

**Figure 5:**
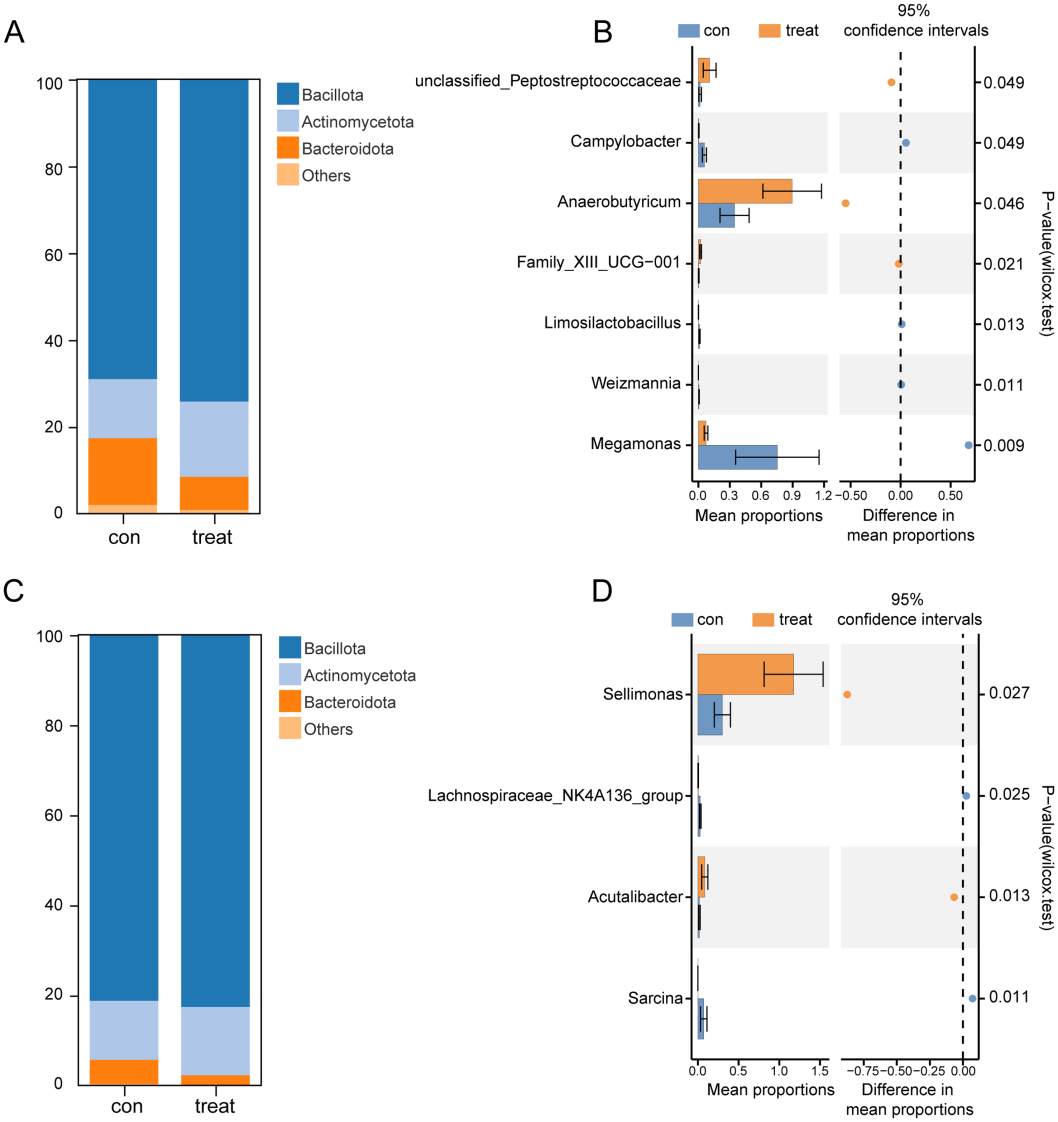
Effects of dietary nucleotide supplementation on bacterial community composition in kittens. **(A,B)** Before dietary treatment; **(C,D)** After dietary treatment.

Before dietary treatment (Figures 5A,B), at the phylum level, the fecal microbial communities of both the CON group and the TREAT group were dominated by *Bacillota*, followed by *Actinomycetota* and *Bacteroidota*. At the genus level, the relative abundances of *unclassified*_*Peptostreptococcaceae*, *Campylobacter*, *Anaerobutyricum*, and *Family_XIII_UCG-001* in the TREAT group were significantly higher than CON group (*p* < 0.05), the relative abundance of *Megamonas* in the CON group was significantly higher than that in the TREAT group (*p* < 0.01). After 42 days of dietary treatment (Figures 5C,D), at the phylum level, the relative abundance of *Bacillota* was further increased in both groups, the relative abundance of *Bacteroidota* was significantly decreased, and *Actinomycetota* remained at a low relative abundance level. At the genus level, compared with the CON group, the relative abundances of *Sellimonas* and *Acutalibacter* in the TREAT group were significantly enriched (*p* < 0.05). The relative abundances of *Lachnospiraceae_NK4A136_group* and *Sarcina* were significantly higher in the CON group and were almost undetectable in the TREAT group (*p* < 0.05).

## 4 Discussion

The results of this study demonstrated that dietary supplementation with 0.03% yeast-derived nucleotides in weaned kittens could significantly improve apparent nutrient digestibility, increase the serum levels of growth hormones and growth-related regulatory factors, attenuate the declined immune function, and exert a positive regulatory effect on the diversity and community structure of intestinal microbiota. This finding provides a scientific theoretical basis for the application of nucleotides as functional nutritional additives in the diet of weaned kittens. Similar regulatory effects have been reported in kittens (16) and other young livestock and poultry, suggesting that dietary nucleotides may exert physiological regulatory effects through the synergistic action of multiple pathways involving the “nutrition-immunity-microecology” axis.

Previous studies reported that weaning stress causes body weight loss and feed intake reduction with the decreased nutrient digestibility as well as diarrhea in the early weaning stage of pigs (17). However, supplementation of dietary nucleotides can mitigate the negative effects induced by weaning stress (18). This study showed that there is no difference in ADG and DMI whether supplemented nucleotides to kittens, but it presented a significantly increase in feline nutrient apparent digestibility after 42 days of nucleotides treatment. Indicated nucleotides are beneficial to improving nutrient utilization efficiency of weaned kittens. Moreover, supplementation of dietary nucleotides also benefited in body condition of kittens with the BCS more closed to the well-proportioned ideal body condition (point 5) compared to control group. For kittens, which are highly sensitive to dietary changes and environmental stress, fecal state and is an important visualized indicators for evaluating intestinal physiological health. Improved fecal status was also observed in the group supplemented of nucleotides, which showed a significant decrease in fecal score and well-formed feces. The results suggest that dietary nucleotides ameliorated the negative effects on weaning stress of kittens. The growth performance indices did not show a statistically significant difference may because of the limited animal samples, short duration of the feeding period, different breed and large individual physiological differences among weaned kittens. Extending the feeding trial period and sample size may lead to statistical differences in growth performance indices, which is consistent with the research results reported in weaned piglets and poultry (19, 20).

The intestinal tract of weaned kittens is still in the developmental stage, with relatively unstable secretion of digestive enzymes, intestinal villus structure, and mucosal barrier function, which are easily affected by dietary components and intestinal microenvironmental factors. During the growing stage which keeps a high-level requirement for protein and DNA synthesis, exogenous dietary nucleotides plays a role in regulating the proliferation of enterocytes and promote intestinal mucosa recovery and enhance the activity of brush-border enzymes (21). Nucleotides are mainly absorbed in the upper region of the small intestine (22), and it is also proved nucleotides may affect enterocyte differentiation and enhance enzyme activity *in vitro* (23). In addition, treat with dietary nucleotides significant improved ileal villus heights and villi crypts depth on chicken (24, 25). In current research, we found a significant difference in apparent digestibility of dry matter, organic matter, crude protein and ether extract in the nucleotide-supplemented group. The results suggested that nucleotide supplementation may improve nutrient absorption efficiency, especially the utilization rate of nitrogenous nutrients such as crude protein, by improving the morphological structure of the intestinal mucosa and promoting the proliferation, renewal, and repair of intestinal epithelial cells (18).

The results revealed that all serum biochemical indicators were within the reference intervals in both groups (26, 27), and no significant differences were observed between two groups. However, TP, ALB, GLB Crea, and AST levels were significantly increased after 42 days of dietary treatment. Variations in ALB levels partially reflect the status of protein digestion, absorption, and metabolism in the animal (28). Hamdi (29) reported the elevated AST level implicated the increased amino acid metabolism and protein synthesis. These results in accordance with the higher CP apparent digestibility, suggesting nucleotide supplementation may had the positive effect on protein turnover of kittens in this study.

Growth hormone (GH) and insulin-like growth factor-1 (IGF-1) are important endocrine regulatory indicators that reflect the growth and development level of the animal body. This study found that the serum concentrations of GH and IGF-1 in kittens in the nucleotide-supplemented group were significantly increased after 42 days of dietary treatment and were significantly higher than control group, which is consistent with previous study that dietary nucleotide supplementation may indirectly enhance protein anabolism, bone development, and body growth potential by activating the activity of the GH/IGF-1 endocrine axis (30). Combined with the significant improvement in apparent crude protein digestibility observed in this study, it can be inferred that nucleotides exert a dual promoting effect on protein nutrition in weaned kittens: on the one hand, nucleotides improve the digestive and absorptive efficiency of dietary crude protein; on the other hand, nucleotides enhance the anabolic utilization of absorbed amino acids in body tissues through the endocrine regulatory pathway. This result is consistent with the “growth-promoting” effect of nucleotides reported in infant formula and young animal diets (5, 8).

The feline serum IgG, IgA, and IgM concentrations in both nucleotide-supplemented and control groups were all in a relative high level at the initiation of experiment, and there is no significant different between two groups. This may because of the vaccine treatment before the experiment. After 42 days feeding treatment, although the subsequent vaccine treat was completed, the immune effect remains declined presented by the significant decrease in serum IgA, and IgM concentrations in both groups. However, previous research showed that kittens supplemented with nucleotides showed significantly improved antibody response to specific vaccine at various timepoints (16). In this study, we found IgG concentration presented a similar trend in nucleotide-supplemented group, which was increased significantly compared to 0 day and higher than control group. Besides, IgA, and IgM concentrations were also significantly higher in nucleotide-supplemented group compared to control group on the 42 days of the experiment. This result suggested that exogenous nucleotide supplementation may effectively enhance the humoral immune response capacity of weaned kittens, which previous research also provided evidence in nucleotide supplementation on boosting immune responses of piglets (5, 31).

Nucleotide supplementation can provide sufficient metabolic substrates for the proliferation and differentiation of immune cells such as lymphocytes and macrophages, which is conducive to improving the efficiency of antibody production and enhancing the immune effect of vaccine. Relevant studies have shown that dietary nucleotide supplementation can increase the serum levels of IgG, IgA, and IgM in kittens and piglets, enhance the humoral immune response (31, 32). Notably, with the extension of the feeding trial period, the serum levels of IgA and IgM in kittens of both groups showed a decreasing trend, but the levels in the nucleotide-supplemented group were still significantly higher than control group. This result may be associated with the immune activation and decline by vaccine. Previous study showed nucleotides supplementation can accelerate the maturation of immune cells and alleviate weaning-induced immune suppression (8). Therefore, dietary nucleotide supplementation may slow down the decline of immune reaction, help kittens maintain a relative high level of immune protection during the immune gap period, and reduce the risk of potential infectious diseases.

Intestinal microbiota is an important component of the host nutrient metabolism and immune regulation system, and plays a crucial role in maintaining intestinal mucosal barrier function and host physiological health. Through 16S rRNA gene sequencing analysis, we found that prior to dietary treatment, the overall alpha diversity and community structure of fecal microbiota were broadly similar between the two groups. After 42 days of dietary treatment, a significant differentiation in beta diversity of the microbial community was observed between the two groups, suggesting that dietary nucleotide supplementation exerts a specific regulatory effect on the overall structure of intestinal fecal microbiota. Similar intestinal microbial flora remodeling effects have been reported in kittens and chicks supplemented with dietary nucleotides (16, 20). The Shannon index and species richness index of the nucleotide-supplemented group were slightly lower than those of the control group; combined with the changes in the number of OTUs, this result indicated that nucleotide supplementation may reduce the complexity of the intestinal microbial flora to a certain extent, but steady the number of shared core OTUs between the two groups, making the intestinal microecological environment more stable and concentrated. Previous study had pointed out that under normal physiological health conditions, a moderate decrease in alpha diversity of intestinal microbiota does not necessarily indicate a decline in microbial functional activity, but may reflect the enrichment of dominant functional microbial flora with beneficial physiological effects (33).

Before and after dietary treatment, the fecal microbiota of both groups was dominated by *Bacillota* at the phylum level, with relatively low relative abundances of *Bacteroidota* and *Actinomycetota*. After 42 days of dietary treatment, the relative abundance of *Bacillota* was further increased and the relative abundance of *Bacteroidota* was significantly decreased in both groups, which may be related to the high animal protein and high fat dietary structure of the basal diet and the regulatory effect of nucleotides on the metabolic substrates of intestinal microbiota. Most microbial members of *Bacillota* are closely associated with the production of short-chain fatty acids (SCFAs) and energy recovery in the intestinal tract, and a moderate increase in their relative abundance is conducive to improving intestinal energy utilization efficiency and maintaining the integrity of the intestinal mucosal barrier (34, 35).

Prior to dietary intervention, alpha diversity indices and overall microbial community structures were generally comparable between the control and experimental groups, indicating that randomization effectively minimized major baseline differences. However, at the genus level, some individual variations were observed: the experimental group showed relatively higher abundances of *unclassified_Peptostreptococcaceae*, *Campylobacter*, and *Anaerobutyricum*, whereas *Megamonas* was enriched in the control group. These baseline differences represent a limitation of the current study and should be considered when interpreting post-treatment comparisons. Longitudinal analyses revealed treatment-specific microbial shifts following dietary supplementation: *Sellimonas* and *Acutalibacter* were enriched in the nucleotide-supplemented group, whereas *Lachnospiraceae_NK4A136_group* and *Sarcina* were enriched in the control group and nearly undetectable in the supplemented group. Despite the baseline individual differences, the overall patterns of microbial response were distinct, suggesting that dietary treatment effects were largely independent of initial variability.

Although functional verification experiments were not conducted in this study, existing literature indicates that these genera are involved in the fermentation of soluble carbon sources, SCFA production, and regulation of intestinal mucosal immunity (29, 30). Based on this, it can be inferred that dietary nucleotide supplementation may selectively promote colonization and proliferation of potentially beneficial microbes while inhibiting the growth of genera potentially associated with intestinal inflammation or diarrhea, likely through modulation of small-molecule metabolic substrates and the gut luminal environment (36). Notably, the temporal dynamics of these microbiota shifts corresponded with observed improvements in nutrient digestibility and immune function, suggesting that intestinal microecology may play a key mediating role in the nutritional and immunoregulatory effects of nucleotides in weaned kittens.

Comprehensive analysis of the results of growth performance, apparent nutrient digestibility, endocrine and immune parameters, and intestinal microbial diversity revealed that the regulatory effects of nucleotides on weaned kittens probably exhibit multi-level and synergistic characteristics: first, nucleotides promote the maturation of intestinal structural and functional development, improve the digestive and absorptive capacity of nutrients, and provide a sufficient material basis for animal growth and development; second, nucleotides activate the GH/IGF-1 endocrine axis and humoral immune response, enhance the body growth driving force and slow down the decline of immune reaction; third, nucleotides regulate the diversity and community composition of intestinal microbiota, optimize the interaction environment between the host and intestinal microbiota, thereby indirectly supporting the host nutrient metabolism and immune homeostasis. These three regulatory aspects interact with each other and are inseparable, which is also consistent with the current theoretical framework of “gut-immune-metabolism” integrated regulation in animal nutrition (37).

This study has certain limitations that need to be acknowledged: first, the feeding trial period was 42 days, which mainly covered the early weaning stage of kittens, and is insufficient to evaluate the long-term regulatory effects of nucleotide supplementation on the growth, development, and health status of kittens throughout the growth period and even in adulthood; second, the sample size of experimental kittens was relatively limited, which may reduce the statistical power to detect significant differences in partial indices; third, the microbial diversity analysis was mainly based on 16S rRNA gene high-throughput sequencing, which has limitations in accurately resolving the microbial community to the species level, and multi-omics technologies such as metabolomics or transcriptomics were not combined to conduct an in-depth analysis of the changes in microbial functional activity and host metabolic pathways. This study only set a single-dose treat group that referenced the previous research (16) due to the limited sample size. Future research can further explore the dose-effect relationship of different doses and sources of nucleotides on the growth, immunity, and metabolism of weaned kittens on the basis of expanding the sample size and extending the feeding trial period. In addition, multi-omics technologies can be combined to deeply reveal the regulatory mechanism of intestinal microbiota in the process of nucleotides exerting their physiological functions (38), so as to provide a more comprehensive and in-depth scientific basis for the rational application of nucleotides in pet feed.

## 5 Conclusion

Supplementation of 0.03% yeast-derived nucleotides in the diet of weaned kittens can significantly improve the nutrient apparent digestibility, enhance serum GH, IGF-1 and IgG levels, attenuated the decline of IgA and IgM. This improvement is likely influenced by intestinal microorganisms, including *Sellimonas* and *Acutalibacter*. Therefore, 0.03% nucleotides supplementation presents a potential to maintain relatively higher humoral immunity and improve health condition of weaned kittens.

## Data Availability

The datasets presented in this study can be found in online repositories. The names of the repository/repositories and accession number(s) can be found at: https://www.ncbi.nlm.nih.gov/, PRJNA1444892.
